# *C*-Terminal Fragment of Vitellogenin II, a Potential Yolkin Polypeptide Complex Precursor Protein—Heterologous Expression, Purification, and Immunoregulatory Activity

**DOI:** 10.3390/ijms22137223

**Published:** 2021-07-05

**Authors:** Agnieszka Szmyt, Agnieszka Zabłocka, Józefa Macała, Józefa Chrzanowska, Anna Dąbrowska

**Affiliations:** 1 Department of Functional Food Products Development, Wroclaw University of Environmental and Life Science, 37 Chełmońskiego Str., 51-640 Wrocław, Poland; a.tatomir.szmyt@gmail.com (A.S.); jozefa.chrzanowska@upwr.edu.pl (J.C.); 2 Department of Microbiology, Hirszfeld Institute of Immunology and Experimental Therapy, Polish Academy of Science, 12 Rudolf Weigl Str., 53-114 Wrocław, Poland; agnieszka.zablocka@hirszfeld.pl (A.Z.); jozefa.macala@hirszfeld.pl (J.M.)

**Keywords:** YGP40, vitellogenin, yolkin polypeptide complex, immunoregulatory activity

## Abstract

The aim of this research was to analyze the heterologous expression, purification, and immunoregulatory activity of recombinant YGP40 (rYGP40), the potential precursor of the yolkin peptide complex. The ygp40 coding sequence was codon optimized, successfully expressed in the *E. coli* system, and purified from inclusion bodies with a yield of about 1.1 mg/L of culture. This study showed that the protein exhibits immunomodulatory activity, expressed by the stimulation of TNF-α and IL-10 production and nitric oxide induction at a level comparable to that of the natural yolkin peptide complex obtained by other authors from hen egg yolk. At the highest dose of 100 µg/mL, rYGP40 also caused the up-regulation of iNOS expression in murine bone marrow-derived macrophages (BMDM). Moreover, no cytotoxic effects of rYGP40 on the BMDM cell line were observed.

## 1. Introduction

Hen egg is a rich source of bioactive proteins, among which an important role is played by vitellogenin [1,2,3,4,5]. This protein is a serum lipoprotein specific to laying hens and is proteolytically cleaved into heavy- and light-chain lipovitellins and phosvitin, the major yolk granule proteins. Another proteolytic product of vitellogenin II (VTG2) is YGP40—a yolk glycoprotein with a molecular weight of 40 kDa. The protein is released by cathepsin D from the *C*-terminal fragment of chicken VTG2 and is a potential source of several peptides with proven immunoregulatory activities [6,7,8]. This polypeptide complex, named yolkin, consists of peptides with molecular masses from 1 to 35 kDa and possesses biological properties similar to the mammalian proline-rich polypeptide complex (PRP) [9]. Yolkin possesses immunoregulatory activity and stimulates human whole blood [7,8,10] and mouse macrophages of the BMDM cell line [11] to produce and release pro-inflammatory factors, such as interferons α/β (IFNs α/β), interleukin 1 beta (IL-1β), interleukin 6 (IL-6), tumor necrosis factor alpha (TNF-α), and nitric oxide, and anti-inflammatory cytokine interleukin 10 (IL-10). Furthermore, the peptide complex also exhibits antioxidant activity, which is fundamental for protection against oxidative damage, and stimulates neuron-like PC12 cells and human whole blood to secrete the mature form of brain-derived neurotrophic factor (BDNF) [10,12]. BDNF, together with IL-1β and IL-6, plays an important role in the control of the central nervous system functionality [13,14,15,16]. Yolkin also moderates aging symptoms and supports learning functions. It was shown that yolkin has a significant influence on behavior and cognitive functions in both young and old rats [17]. It seems that the yolkin polypeptide complex may also have a positive therapeutic effect on human neurodegenerative diseases, such as Alzheimer disease.

The natural source of yolkin is the yolk of a laying hen’s egg. The methods applied for its isolation [8,10,18] allow one to obtain a heterogeneous group of yolk-derived peptides, alongside IgY purification; nevertheless, the purification yield of both methods is not high [8]. Nowadays, another approach to produce different biopeptides involves recombinant DNA technology. This alternative way allows their heterologous expression in a host organism of different origin. The method is based on the introduction of a DNA fragment that encodes a peptide or protein fragment into a host cell integrated with a suitable carrier—most frequently plasmid DNA. The genetically modified organisms are induced for production of heterologous peptides or proteins; most frequently, bacteria such as *Escherichia coli* are used for this purpose. For the expression of more complex molecules, such as those that are glycosylated or those with a domain structure, cells of higher organisms are used, such as yeast, filamentous fungi, insect cells, or specialized cell lines derived from the tissues of higher eukaryotes. The advantage of prokaryotic expression systems lies in their well-known genetics, a wide selection of many elaborate molecular tools capable of producing high yields of expression in a short time. These methods are also relatively easy to use, and their costs are lower in comparison to, e.g., cell lines. In addition, this technology allows a quick introduction of sequence modifications so that it is possible to evaluate multiple variants and quickly change (strengthen or weaken) their biological activity. In our studies, we used it for the heterologous expression of YGP40 cds, which was further analyzed for its immunological activity on the cellular model of mouse bone marrow-derived macrophages (BMDM). It is well known that macrophages are key cells of the innate immune system responsible for tissue homeostasis. Those cells are actively involved in the primary immune response to pathogens, such as bacteria or viruses, and also in neurodegenerative disorders [19,20]. As antigen-presenting cells, they help to form the innate and adaptive immune responses by producing and releasing a wide spectrum of pro-inflammatory mediators. Activated macrophages produce pro-inflammatory factors, such as type one interferons (IFNs), tumor necrosis factor alpha (TNF-α), or inducible NOS-dependent nitric oxide (NO), and also anti-inflammatory and immunoregulatory factors, such as interleukin 10 (IL-10) and transforming growth factor beta (TGF-β) [20,21]. It was previously shown that the yolkin polypeptide complex stimulates the mouse macrophages of J774 and BMDM cell lines to produce and release significant amounts of type one interferons, tumor necrosis factor alpha (TNF-α), and nitric oxide [7,10,11,18].

The aim of the current research was the production of the potential recombinant precursor of yolkin peptides (rYGP40) in the *Escherichia coli* BL21 expression system, its purification, and analysis of its immunoregulatory activity expressed as the ability to stimulate whole blood cells and BMDM macrophages to the secretion of cytokines TNF-α and IL-10. Moreover, the impact of rYGR40 on the expression of inducible nitric oxide synthase (iNOS) and the production of nitric oxide by BMDM macrophages was determined. This study is the first report in which YGP40 is efficiently produced using a bacterial system and reveals high immunostimulatory activity in in vitro assays on human whole blood and murine bone marrow-derived macrophages (BMDM).

## 2. Results

### 2.1. Cloning of ygp40 Gene

The heterologous expression of genes in *E. coli* is an alternative tool for obtaining selected proteins, when isolation from natural sources is complicated or of low yield. As yolkin isolation from hen eggs gives a preparation of diverse activity and peptide composition, our goal was to clone its precursor’s coding sequence (YGP40) and obtain it in a prokaryotic expression system.

To achieve high heterologous expression of rYGP40 in *E. coli*, the *C*-terminal fragment of the chicken ygp40 gene sequence was *E. coli* codon-optimized without modifying the protein sequence and synthesized by Gene Art (Germany) and cloned to the pQE80L expression vector, which allowed the production of a recombinant protein with an *N*-terminal His-Tag. The gene sequence analysis showed 100% identity with the ygp40 DNA sequence synthesized by GeneArt. After verification, the construct pQE80L/ygp40 was successfully transformed into *E.coli* BL21 (DE3) expression cells.

### 2.2. Expression and Solubility Analysis of rYGP40

**Figure 1:**
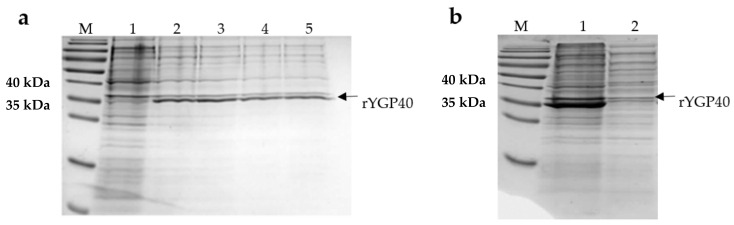
(**a**) rYGP40 expression analysis by SDS-PAGE. 1: sample taken before the expression induction with IPTG, lanes 2–5: samples taken after 1–4 h after expression induction. (**b**) rYGP40 solubility analysis; 1—pellet/insoluble fraction; 2—supernatant/soluble fraction. M: protein weight marker PageRuler Prestained Protein Ladder.

*E. coli* BL21 (DE3) transformed with pQE80L/ygp40 efficiently produced the rYGP40 protein. The band, of approximately 37 kDa, corresponding to the molecular weight of the recombinant protein, was observed on Coomassie R-250-stained polyacrylamide gel, which appeared after IPTG induction. Its intensity increased with time during the expression at 37 °C. (Figure 1a). The solubility analysis of rYGP40 showed that the protein was predominately identified in the insoluble fraction as inclusion bodies (Figure 1b). The attempts to obtain the rYGP40 in a soluble form (lower expression temperatures, lower concentration of inducer, cloning to another vector, and autoinducing medium) did not improve its solubility—data not shown. Thus, in our study, we used the pQE80L/ygp40 construct for the expression of recombinant YGP40 with *N*-terminal His-Tag and focused on improving the expression conditions for efficient inclusion bodies isolation. After optimization, the expression was held on LB medium at 37 °C for 6h and with a final 1 mM concentration of IPTG. The optimized parameters resulted in a high amount of recombinant protein accumulated in inclusion bodies. 

### 2.3. Inclusion Bodies Isolation and rYGP40 Purification

**Figure 2:**
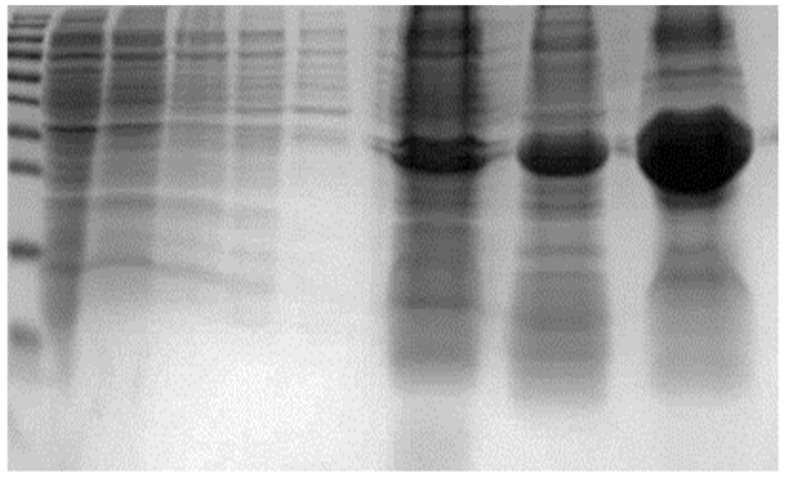
IBs of rYGP40 isolated from *E. coli* BL21 (DE). M—molecular marker: PageRuler Prestained Protein Ladder; 1: biomass wash with 0.9% NaCl; 2a, 2b: biomass wash with TTN buffer; 3a, 3b: biomass wash with TN buffer; 4: biomass sample after 6h of expression; 5, 6: isolated IBs of rYGP40.

The isolation of IBs was carried out from 2L of culture. The applied isolation technique allowed one to obtain approximately 1 g of crude inclusion bodies from about 5 g of wet cell biomass. The isolated aggregates were made mostly of rYGP40 protein (Figure 2). Although strong denaturing conditions had to be applied to solubilize the isolated inclusion bodies fraction, still a significant part of recombinant protein remained insoluble in isolated precipitates.

**Figure 3:**
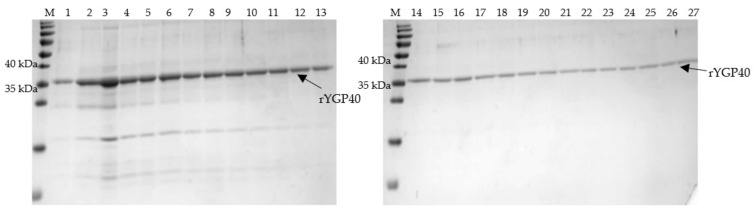
SDS-PAGE analysis of fractions eluted from Talon resin after incubation with solubilized IBs of rYGP40; M—molecular marker: PageRuler Prestained Protein Ladder; lanes 1–27: elution fractions (20 µL of each 0.5 mL fraction were loaded on gel).

The solubilized fraction of IBs was used for IMAC on Talon resin. The purification process was conducted under denaturing conditions in the presence of 8M urea, as our previous isolation experience showed that application of decreasing urea concentration during the purification procedure for gradual refolding of the protein resulted in premature protein release from the Talon resin. Hence, it was necessary to keep the conditions as described above to maintain the rYGP40 in its denatured form and enable the exposed His-Tag to bind to the column bed. Subsequently, the protein was released from the column by the pH drop from 8.9 to 4.5 instead of the imidazole-dependent elution. A single band corresponding to rYGP40 was detected in eluted fractions (Figure 3), which were pooled for further processing.

**Figure 4:**
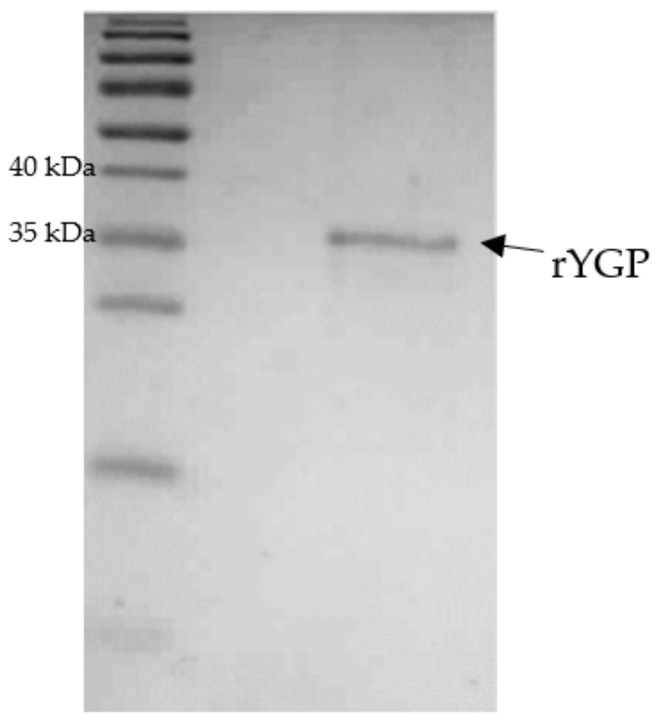
Electrophoretic analysis of rYGP40 after purification and 10× concentration on Amicon: M—molecular marker: PageRuler Prestained Protein Ladder; rYGP40—recombinant YGP40.

The renaturation of rYGP40 was conducted by a rapid dilution method in non-denaturing buffer with nonionic polyoxyethylene surfactant. The detergent was added for stabilization of the refolded protein. After overnight incubation at 4 °C, the protein suspension was concentrated with an Amicon 30 kDa cut-off membrane, and its purity was verified on SDS-PAGE. The obtained results indicated that rYGP40 was a homogenous preparation. The electrophoretic analyses revealed the presence of one band corresponding to approximately 37 kDa as shown in Figure 4. The recombinant YGP40 concentration was determined by the Bradford method [22] to be 150 µg/mL; thus, the isolation and purification yield was 1.125 g of the recombinant protein/1 L of the bacterial culture. It was determined that rYGP40 preparation is free of bacterial endotoxins.

### 2.4. Impact of rYGP40 Preparation on BMDM Cells Viability

**Figure 5:**
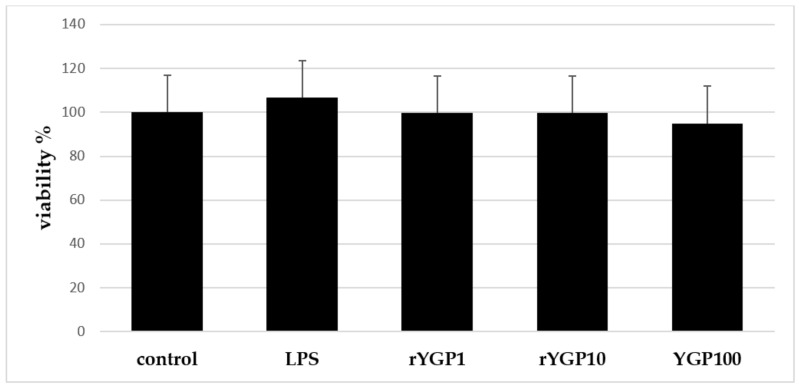
Effect of rYGP40 preparation on viability of bone marrow-derived macrophages of BMDM cell line. BMDM cells (1 × 10^5^/mL) were exposed to rYGP40 at doses of 1, 10, and 100 μg/mL (rYGP 1, 10, and 100) for 24 h at 37 °C at 5% CO_2_. LPS (1 μg/mL) was used as a positive inducer. Cell viability was evaluated by MTT assay. The data represent means ± SD of four independent experiments.

According to MTT assay results, it was shown that rYGP40 preparation is not toxic for BMDM cells at doses applied in the experiment: 1–100 µg/mL (Figure 5).

### 2.5. Impact of rYGP40 Preparation on TNF-α and IL-10 Production

**Figure 6:**
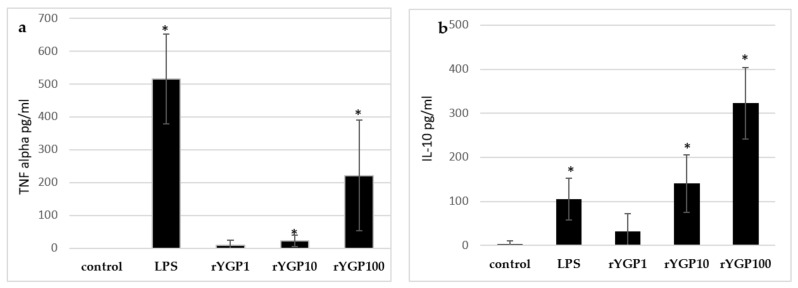
Production of TNF-α (**a**) and IL-10 (**b**) by human whole blood in response to rYGP40 treatment. The data represent means ± SD of four independent experiments. * *p* ≤ 0.05 vs. control.

**Figure 7:**
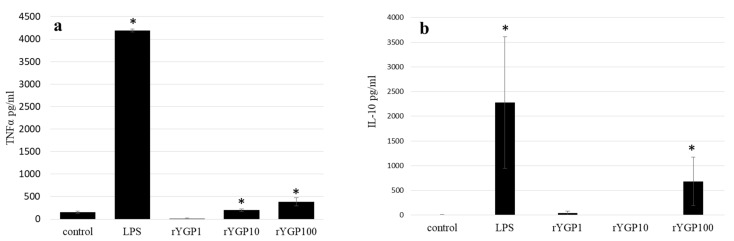
Production of TNF-α (**a**) and IL-10 (**b**) by BMDM cells in response to rYGP40 treatment. The data represent means ± SD of four independent experiments. * *p* ≤ 0.05 vs. control.

To determine whether rYGP40 preparation activates TNF-α and IL-10 expression and production, whole blood cells or murine macrophage BMDM cells were treated with rYGP40 at doses ranging from 1 to 100 µg/mL. It was observed that rYGP40 significantly increased the expression and production of cytokines in both human whole blood and BMDM macrophages: pro-inflammatory TNF-α at doses of 10 and 100 μg/mL in both whole blood (Figure 6a) and BMDM cells (Figure 7a) and anti-inflammatory IL-10 at doses of 10 and 100 μg/mL in whole blood (Figure 6b) and only at 100 μg/mL in BMDM cells (Figure 7b).

One milliliter portions of human whole blood were treated with inducers: rYGP40 at doses of 1, 10, and 100 µg/mL (rYGP1, 10, 100) or LPS (1µg/mL) used as a positive control, for 24 h at 37 °C. Control samples containing non-treated blood cells were used to measure spontaneous production of cytokines. Supernatants were collected, and the levels of TNF-α and IL-10 were determined by ELISA. Results are presented as mean ± SD (*n* = 4). * *p* ≤ 0.05—statistically significant difference in value versus control.

BMDM cells (1 × 10^6^/mL) were either treated with rYGP40 at doses of 1, 10, and 100 µg/mL (rYGP1, 10, 100) or LPS (1µg/mL) used as a positive control, for 24 h at 37 °C. Non-treated BMDM cells were used as a negative control. Supernatants were collected, and levels of TNF-α and IL-10 were determined by ELISA. Results are presented as mean ± SD (*n* = 3–4). * *p* ≤ 0.05—statistically significant difference in value versus control.

### 2.6. Impact of rYGP40 Preparation on Nitric Oxide Production and iNOS Expression

**Figure 8:**
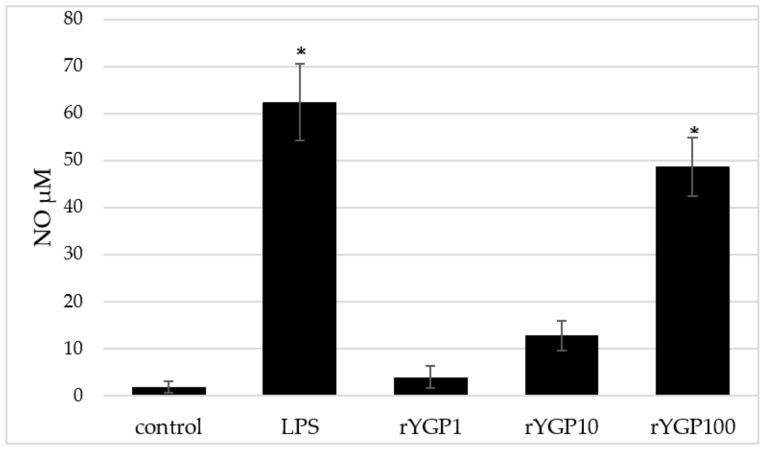
Effect of rYGP40 on nitric oxide production in BMDM cells. The data represent means ± SD of four independent experiments. * *p* ≤ 0.05 vs. control.

**Figure 9:**
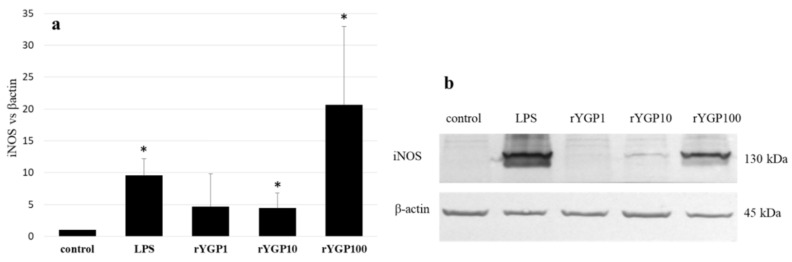
Up-regulation of iNOS expression in BMDM cells after rYGP40 treatment. The level of iNOS protein was analyzed in cell lysate in comparison to β-actin (**a**) by immunoblotting (**b**) using monoclonal anti-iNOS antibodies. The data represent means ± SD of four independent experiments. * *p* ≤ 0.05 vs. control.

rYGP40-treated BMDM macrophages showed a significant, dose-dependent, NO production increase (Figure 8). Application of rYGP40 to the cells from 1 to 100 µg/mL resulted in a significant increase in the level of released NO (*p* ≤ 0.05), from 4 to 49 µM, respectively. Moreover, a significant impact of rYGP40 on the up-regulation of iNOS expression was shown. As shown in Figure 9, an approximately 20-fold increase was observed in the iNOS level in response to rYGP40 compared to the control cells.

BMDM cells (1 × 10^6^/mL) were treated with rYGP40 at doses of 1, 10, and 100 µg/mL (rYGP1, 10, and 100) or LPS (1 µg/mL) used as a positive control, for 24 h at 37 °C. Non-treated BMDM cells were used as a negative control. Results are presented as mean ± SD (*n* = 6). * *p* ≤ 0.05—statistically significant difference in value versus control.

BMDM cells were treated with rYGP40 at doses of 1, 10, and 100 µg/mL (rYGP1, 10, and 100) or LPS (1 μg/mL) used as a positive control, for 24 h at 37 °C. Non-treated BMDM cells were used as a negative control. The level of iNOS protein was detected in cell lysate by immunoblotting using monoclonal anti-iNOS antibodies (9b). Fold change in iNOS levels compared to β-actin (9a). Results represent 3–4 independent experiments and data are presented as mean ± SD. * *p* ≤ 0.05—statistically significant difference in value versus control.

## 3. Discussion

This investigation demonstrates the heterologous expression of YGP40 protein as a potential precursor of the yolkin peptide complex and the analysis of its immunoregulatory activity. Under physiological conditions, the protein is obtained by proteolytical cleavage of vitellogenin II by cathepsin D, during or after transportation into oocyte. During the process, the major yolk granule proteins are also produced [6,23]. Yamamura et al. [6] showed that the amino acid sequence of the 40 kDa glycoprotein corresponds with the *C*-terminal fragment of vitellogenin II. YGP40 is composed of 284 amino acid residues located between Ala-1567 and Thr-1850 of the vtg II amino acid sequence; however, to date, its biological activity has not been investigated.

In the research of Polanowski et al. [7], it was shown for the first time that hen egg yolk immunoglobulin Y, γ-livetin of yolk plasma, is accompanied by a polypeptide complex, named yolkin. The analysis of the *N*-terminal amino acid sequences of eight of the electrophoretically separated yolkin constituents shows that all of them are homologous with fragments of the *C*-terminal vitellogenin II domain YGP40 protein. A part of the yolkin fractions (MW of about 4 and 12 kDa) are carbohydrate free and are complementary to vitellogenin II starting from the residue 1732 in its amino acid sequence, while the other fractions (with MW of about 16, 19, 23, 29, 32, and 35 kDa) appear to be glycoproteins corresponding to the amino acid sequence of vitellogenin II at 1572 amino acid residue [8,24]. Therefore, it was speculated that yolkin constituents may be considered as a set of peptides obtained as a result of proteolytic cleavage of the YGP40 at positions 1571S-1572A and 1731R-1732M [8].

Further research on yolkin showed that the peptide complex exhibits significant immunoregulatory activity. Yolkin peptides isolated from natural sources stimulated human whole blood to release significant amounts of cytokines, both pro-inflammatory, such as IL-1β, TNF-α, or IL-6, and anti-inflammatory, such as IL-10 [7,8]. Yolkin also up-regulated the expression of inducible nitric oxide synthase (iNOS) and increased the production of nitric oxide (NO), TNF-α, and type I IFNs (α/β) by bone marrow mouse macrophages of the BMDM cell line, which triggered antiviral activity [11]. The complex enhances the behavioral and cognitive functions of the brain, which was shown by Lemieszewska et al. [17] in an animal model.

These data indicate that yolkin affects the regulation of the immune system and stimulates the antiviral response; therefore, it can be used as an effective immunostimulator of innate immunity or a supplement of conventional therapy for immunodeficiency. Yolkin may be also associated with effects on the cells of the central nervous system and also on peripheral blood cells entering the brain through a defective blood–brain barrier. These cells are stimulated by the production and secretion of substances with neuroprotective or/and regulatory effect on neurons; therefore, yolkin may find application in prevention and treatment of aging-related neurodegenerative disorders.

Although the immunoactive properties of yolkin peptides are confirmed, there are no data indicating the possible biological activity of YGP40 as a potential precursor of the yolkin polypeptide complex. The analysis of yolkin peptide activity is performed after the isolation of the complex from hen eggs, when the natural processing of YGP40 has already proceeded.

Therefore, our goal was to obtain YGP40 as the recombinant protein (rYGP40) and analyze its immunoregulatory activity.

There are multiple pieces of evidence that demonstrate that increased cytokine production/secretion can be used as an indicator of immunological status of immunocompetent cells [25]. Therefore, the impact of rYGP40 on cytokine induction under ex vivo stimulation of human whole blood was determined. The chosen method enables one to keep the natural microenvironment and communication/interaction between different blood cell populations [26,27]. Two types of cytokines were studied—pro-inflammatory TNF-alpha secreted by Th1 cells, which control the cellular immunity, and anti-inflammatory cytokines, such as IL-10 secreted by Th2 cells, which are responsible for the control of the humoral immune response. The obtained results showed that rYGP40, at the dose of 100 µg/mL, up-regulated the production of both pro-inflammatory TNF-alpha and anti-inflammatory IL-10. This indicates that rYGP40 exhibits immunoactive properties, and its cytokine-inducing activity is comparable to that of the yolkin polypeptide complex isolated from natural sources [8,28].

Macrophages play pivotal roles in modulating the function of the immune system. They maintain homeostasis, secrete numerous factors responsible for the regulation of other cell functions, and protect against viral and bacterial infections [24,29]. The major mediators of macrophage effector function, representing the pro-inflammatory M1 macrophage profile, are the generation of cytokines, such as IFNs, IL-1β, or TNF-α; chemokines; reactive oxygen species (ROS); and also nitric oxide (NO).

Results obtained in the present study indicate that rYGP40 is not toxic to the macrophage cells and is able to regulate their immunological activity. The recombinant protein dose-dependently activated BMDM macrophages to produce and secrete pro-inflammatory TNF-α, one of the crucial factors in regulation of the first defense line in innate response, and also the anti-inflammatory IL-10, which is crucial to control/down-regulate inflammation. Both cytokines are important to keep immunological balance. Additionally, rYGP40 is able to up-regulate iNOS expression and NO production, which is comparable to that of the yolkin polypeptide complex isolates from the plasma of egg yolk [11].

The immunostimulatory activity of rYGP40 proves that this protein is biologically active. It is important to underline that recombinant YGP40 obtained in the *E. coli* expression system, in contrast to the originally vitellogenin-derived YGP40 peptide, is not glycosylated. It is known that for some proteins, glycosylation is essential for their biological activity [30]. In some cases, the level of protein glycosylation can increase biological maintenance [31] as was observed for IgY, a main class of avian immunoglobulins [32]. However, Zambrowicz et al. [27] showed the inverse dependence of glycosylation of the yolkin complex on nitric oxide production by BMDM macrophages. Free of carbohydrates, yolkin fractions showed significantly higher activity than their glycosylated equivalents. Moreover, no significant impact on cytokine induction was observed for deglycosylated yolkin fractions compared to the glycosylated ones. Recombinant YGP40 is free of carbohydrate moieties, as *E.coli* does not exhibit glycosylation activity, but the ability of the protein to up-regulate iNOS expression and NO production by BMDM macrophages and stimulate human whole blood to produce cytokines is on a similar level to that of the yolkin complex. This suggests that the presence of carbohydrates is not a critical factor determining the biological activity of YGP40.

For the first time, these results present that YGP40 and a potential precursor of the yolkin polypeptide complex possess immunoregulatory activity comparable to that of the yolkin complex isolated directly from the yolk of a hen egg.

## 4. Materials and Methods

### 4.1. Materials

High-glucose Dulbecco’s modified Eagle medium (DMEM), RPMI 1640 medium, and phosphate-buffered saline (PBS) (pH 7.4) were sourced from the Laboratory of General Chemistry of the Institute of Immunology and Experimental Therapy, PAS (Wrocław, Poland). Bacterial lipopolysaccharide (LPS) from *E. coli* (serotype 055:B5), 3-(4,5-dimethylthiazol-2-yl)-2-5-diphenyltetrazolium bromide (MTT), and Tween 20 were purchased from Sigma (St. Louis, MO, USA). L-glutamine and antibiotics (penicillin/streptomycin mixture) were purchased from BioWest (Nuaillé, France). Reagents for the SDS-PAGE and protein marker were purchased from Bio-Rad (Hercules, CA, USA). Mouse TNF-α and IL-10 ELISA Max™ Deluxe Kit were obtained from BioLegend (San Diego, CA, USA). *N*-(1-naphthyl)-ethylenediamine was purchased from Serva Feinbiochemica (Heidelberg, Germany). Sulfanilamide, sodium nitrite, orthophosphoric acid, acetone, KH_2_PO_4_, and K2HPO4 were purchased from Avantor (Gliwice, Poland). Alkaline phosphatase-conjugated anti-rabbit IgG antibody was purchased from Cell Signaling Technology (Danvers, MA, USA). Anti-iNOS monoclonal antibody was from Santa Cruz Biotechnology (Santa Cruz, CA, USA). 5-Bromo-4-chloro-3-indolyl phosphate disodium salt (BCIP) and nitro blue tetrazolium (NBT) were purchased from Carl Roth GmbH (Karlsruhe, Germany). Restriction enzymes (BamHI, HindIII); kits for the isolation of plasmid DNA, DNA from agarose, and DNA from the PCR reaction; and the protein and DNA markers GeneRuler 1 kb Plus DNA Ladder and PageRuler Prestained Protein Ladder were purchased from Thermo Scientific. Taq DNA polymerase 5 U/μL, 10 mM dNTP, and other PCR reagents were supplied by Eurx. Culture media components (yeast extract, peptone) and reagents such as NaCl, KCl, β-mercaptoethanol, Coomassie Brilliant Blue R-250, Tris-HCl, Triton X-100, and BSA were purchased from Merck and IPTG, urea, and Brij^®^ 35 from Sigma-Aldrich.

### 4.2. Synthesis of ygp40 cDNA

The protein sequence of YGP40 is a *C*-terminal fragment of vitellogenin II (VTG2). The DNA sequence (867 bp) encoding the YGP40 protein was obtained by back-translation of the VTG2 *C*-terminal amino acid sequence (GeneBank CAA31942.1) starting from residue 1572 to 1850. The synthesis of cDNA was performed by GeneArt (Germany). The sequence was optimized for *E. coli* expression, and, for cloning BamHI and HindIII, restriction sites were added. The synthesized sequence was ligated by GeneArt to the pMA-T vector (pMA/ygp40).

### 4.3. Cloning of ygp40 cds to pQE/80L Expression Vector

The pQE80L/ygp40 construct was obtained by ligation of BamHI and HindIII digested fragments of the ygp40 (867 bp) and pQE80L expression vector (4751 bp). The construct was sequenced with primers (ygp40 FCTCGGATCCGCAGAAGCACC-3′) and (ygp40 Rev: 5′-ACCAAGCTTCTTTTAGGTGCTACATTC-3′), and after sequence verification by sequencing (Genomed, Warsaw, Poland), the heat-shock method was used for *E. coli* BL21 (DE3) transformation [33].

### 4.4. PCR Conditions

The identification of ygp40 cds was performed by colony PCR: 95 °C for 2 min; (94 °C for 1 min; 56 °C for 45 s; 72 °C for 45 s) 35 cycles; 72 °C for 10 min.

### 4.5. Overexpression of rYGP40 Protein

The *E. coli* BL21 (DE3) cells, after heat-shock transformation with the pQE80L/ygp40 construct, were plated on LB agar plates with 100 µg/mL of ampicillin and incubated for 18 h at 37 °C. The grown colonies were analyzed by PCR with specific primers (ygp40 For and ygp40 Rev), as described above, to confirm the presence of ygp40 cDNA. For small-scale protein expression analysis, 3 mL of the LB medium with 100 µg/mL of ampicillin was inoculated with a single colony and cultured ON at 37 °C with shaking (180 rpm). The ON culture was used for inoculation (1:100 *v*/*v*) of 5 mL of fresh LB medium. The cells were cultured with shaking (200× rpm) to OD600 nm = 0.4–0.6, and the expression was started by the addition of isopropyl β-D-1-thiogalactopyranoside (IPTG) to the final concentration of 1 mM. Cultures were grown for 4 h, with sample collection before the addition of IPTG and every one hour after the addition of IPTG. Samples were centrifuged for 30 s at 20,000× *g*, and the collected pellets were suspended in either of the following:

- A total of 60 µL of lysis buffer I (20 mM Na_2_HPO_4_, 2 mM KH_2_PO_4_, 150 mM NaCl, 3 mM KCl, and 1 mM β-mercaptoethanol) and analyzed electrophoretically [34] for the detection of recombinant YGP40;
- A total of 500 µL of lysis buffer II (50 mM Na_2_HPO_4_, 300 mM NaCl, 10 mM imidazole, pH 8.0) for recombinant YGP40 solubility analysis.

For large-scale expression, all the performed steps were as described, except the volume culture, which was 2 L, and the expression was held for 6 h at 37 °C with vigorous shaking (220× rpm). The cells were centrifuged at 5500 rpm for 10 min and frozen at −20 °C for further processing—inclusion bodies isolation.

### 4.6. The Analysis of Recombinant YGP40 (rYGP40) Solubility

Bacterial pellets obtained after 4 h of expression (as described above) were suspended in 500 µL of lysis buffer II, sonicated on ice (100% amplitude, 0.75 cycle, 6 min, Sonicator Hielsher UP1000H with MS2 probe), and centrifuged for 10 min at 20,000× *g*. The obtained supernatant, containing the soluble fractions, was transferred into a fresh 1.5 mL Eppendorf tube, while the pellet with the insoluble fraction was re-suspended in 500 µL of lysis buffer II. The samples were analyzed electrophoretically according to that of Laemmli [34].

### 4.7. Electrophoretic Separation under Denaturing Conditions (SDS-PAGE)

The protein electrophoresis under denaturing conditions (SDS-PAGE) was performed according to that of Laemmli [22] on a 6% stacking and 12.5% resolving gel. Analyzed samples with about 20 µg of protein were mixed with 5× of Laemmli sample buffer and incubated at 99 °C for 10 min. The electrophoresis was run at 60 V for 30 min and at 120 V for a further 1.5 h. The gel was stained with Coomassie Brilliant Blue R-250. The Page Ruler Prestained Protein Ladder (Thermo Scientific) was used as a molecular weight marker.

### 4.8. Isolation of Inclusion Bodies (IBs)

Inclusion bodies (IBs) were isolated according to that of Kumar and Krishnaswamy [35] with modifications. Bacterial cells harvested from 2 L of culture were suspended in 0.8% sterile NaCl, sonicated on ice for 6 min (100% amplitude, 0.75 cycle, Sonicator Hielsher UP1000H with MS2 probe), and centrifuged for 10 min at 20,000× *g* and 4 °C. The obtained pellets were washed two times with 40 mL of TTN buffer (50 mM Tris-HCl, 100 mM NaCl, 2% Triton X-100, pH 8.5) and two times with TN buffer (50 mM Tris-HCl, 100 mM NaCl, pH 8.5). The IBs pellet was collected by centrifugation for 20 min at 20,000× *g* and 4 °C and resuspended in 40 mL of denaturing buffer (100 mM phosphate buffer, 8 M urea, 100 mM NaCl, pH 8.0) by overnight mixing with 220 rpm at RT. The insoluble fraction of the IBs was harvested by centrifugation for 20 min at 20,000× *g* and 4 °C, while the solubilized fraction (supernatant) was used for recombinant protein purification.

### 4.9. Recombinant Protein Purification

Recombinant YGP40 purification was performed using ion metal affinity chromatography (IMAC) on Talon resin under denaturing conditions. The soluble fraction of the IBs (supernatant) was used for an overnight batch purification protocol. Four milliliters of Talon resin was equilibrated with denaturing buffer and mixed with solubilized IBs. After overnight incubation with shaking (220× rpm at RT), the suspension was centrifuged at 2500× rpm for 5 min at RT. Afterwards, the resin was washed three times with denaturing buffer, twice with 10× resin volume, and once with 5× resin volume and transferred into a gravity flow column. The recombinant protein was eluted with elution buffer (100 mM phosphate buffer, 8 M urea, 100 mM NaCl, pH 4.5). For elution, 5× resin volume was used. The 0.5 mL fractions were collected and analyzed on SDS-PAGE.

### 4.10. Recombinant Protein Folding and Concentration

The eluted fractions containing the rYGP40 analysis protein were pooled, and the obtained protein solution was diluted 10 times with renaturation buffer (100 Mm phosphate buffer, 100 mM NaCl, 10% *v*/*v* glycerol, 2% *v*/*v* Brij^®^ 35, Sigma Aldrich) (50 mM Tris-HCl of pH 8.5; 100 mM NaCl, 0.2% polyethylene glycol lauryl ether (Brij^®^ 35, Sigma Aldrich), 10% glycerol) to eliminate the influence of 8 M urea. The renaturation process was carried out overnight at 4 °C with gentle mixing. The protein solution was concentrated 10 times on Amicon^®^ Ultra-4 10 K Centrifugal Filter Devices (Merck Millipore), dialyzed against water, and lyophilized.

### 4.11. Determination of Protein Content

The concentration of the recombinant protein was determined with the Bradford method [24], with bovine serum albumin (Sigma) as a standard.

### 4.12. Cell Cultures

#### 4.12.1. Whole Blood Samples

Two milliliter blood samples from healthy donors were kindly provided by the Station of Blood Donation, 4th Military Hospital, Wroclaw, Poland. Samples were collected in syringes containing 10 U/mL of sodium heparin. Within 1 h after the collection, the blood was diluted 10-fold with RPMI 1640 medium supplemented with penicillin/streptomycin and 3% l-glutamine. Whole blood samples were used for the determination of cytokines.

#### 4.12.2. Murine Bone Marrow-Derived Macrophages BMDM

The murine bone marrow-derived macrophages of the BMDM cell line were purchased from Bai Resources. The cells were maintained in Dulbecco’s modified Eagle medium (DMEM) containing 10% fetal bovine serum, antibiotics (penicillin, streptomycin and gentamycin), and 3% L-glutamine. Cells were grown under standard conditions in a humidified incubator at 37 °C in an atmosphere of 95% air and 5% CO_2_.

### 4.13. Assay for Cell Viability

Cell viability was determined using an MTT colorimetric assay [36]. BMDM cells were seeded onto a 96-well plate (1 × 10^4^/well) and incubated for 24 h with rYGP40 preparation (1–100 μg/mL). After cell treatment, the supernatant was removed, and the cells were incubated with MTT (5 mg/mL) for 4 h at 37 °C. The formazan crystals were dissolved by adding 100 µL of DMSO and vigorously shaking to complete resolving. The absorbance was measured by an EnSpire™ 2300 microplate reader (Perkin Elmer, Waltham, MA, USA) at 570 nm. Cell viability was expressed as a percentage of control.

### 4.14. Assay to Nitrite/Nitrate Generation

BMDM cells were plated onto a 24-well plate at a density of 1 × 10^6^ cells/mL and cultured in Dulbecco’s modified medium. rYGP40 preparation (1–100 µg/mL) was added to the cells as a potential inducer of nitric oxide. LPS (1 µg/mL) was used as a positive control, while untreated BMDM cells were used as a negative control. After 24 h of incubation, the supernatants were collected, and the level of nitric oxide was determined.

### 4.15. Assay to Measure Nitric Oxide (NO) Production

NO production was measured by testing the nitrite concentration in the supernatants of cultured BMDM cells using a colorimetric method with Griess reagent [37]. In brief, 100 µL samples of cell supernatants were incubated with an adequate amount of Griess reagent (0.1% *N*-(1-naphthyl)-ethylenediamine and 1% sulfanilamide in 5% phosphoric acid). After 10 min of incubation at room temperature, the absorbance at 550 nm was measured. Levels of nitrite were extrapolated using the sodium nitrite standard curve.

### 4.16. Assay for TNF-α and IL-10 Secretion

#### 4.16.1. Human Whole Blood

Cytokine induction was determined according to the method described by Inglot et al. [25]. One milliliter portions of the blood cell suspension were distributed in 24-well flat-bottomed tissue culture plates. rYGP40 in doses of 1, 10, and 100 µg/mL was used for the analysis. As a positive control, lipopolysaccharide from *E. coli* (serotype: O55:B5) at a dose of 1 ug/mL was used. Control cells containing non-treated blood cell samples (negative control) were used to measure the spontaneous secretion of cytokines. The cells were incubated for 22 h at 37 °C in a 5% CO_2_ atmosphere. The supernatants were used to measure the level of TNF-α and IL-10 by ELISA.

#### 4.16.2. Murine Bone Marrow-Derived Macrophages BMDM

BMDM cells (1 × 10^6^/mL) were distributed into 24-well flat-bottomed tissue culture plates and cultured overnight in Dulbecco’s modified culture medium. Then, cells were treated with rYGP40 preparation at doses ranging from 1 to 100 µg/mL. LPS (1 µg/mL) was used as a positive control. Control cells containing non-treated BMDM cells (negative control) were used to measure the spontaneous secretion of cytokines. After stimulation, the level of TNF-α and IL-10 in the supernatants was determined by ELISA.

### 4.17. Measurement of TNF-α and IL-10 Levels by ELISA

TNF-α and IL-10 secreted from human blood cells and BMDM cells were determined in supernatants by an enzyme-linked immunosorbent assay (ELISA) using human TNF-alpha, human IL-10, mouse TNF-alpha, and mouse IL-10 ELISA Max™ Deluxe Kits (BioLegend, San Diego, CA, USA) according to the procedure recommended by the manufacturer.

### 4.18. Western Blotting

#### 4.18.1. YGP40 Detection

Proteins were electrotransferred to a nitrocellulose membrane (Whatman) with Trans-Blot Cell (BioRad) at 60 V for 2 h. After the transfer, the membrane was blocked with 5% skim milk and TBS buffer overnight at 4 °C. The blocked membrane was incubated with RGS-His antibody (Qiagene) diluted 1:1000 for 1 h at RT, and then washed 5 times for 5 min with TBS-T buffer (TBS buffer with 0.05% Tween 20). In the next step, the membrane was incubated with mouse IgG anti-RGS (H)4 antibody (diluted 1:20,000) conjugated with alkaline phosphatase (Promega) for 1 h in RT and washed as described above. Immunocomplexes were visualized using a BCIP/NBT substrate.

#### 4.18.2. iNOS Detection

BMDM cells (1 × 10^6^ cells/mL) were seeded onto poly-L-lysine-coated 6-well culture plates and incubated with rYGP40 preparation (1–100 µg/mL) or LPS (1 µg/mL) for 24 h for iNOS expression. Next, the cells were lysed in RIPA buffer (150 mM NaCl, 50 mM Tris-HCl pH 7.5, 5 mM EDTA, 1% Triton X-100, 0.1% SDS, 0.5% deoxycholate) supplemented with a protease inhibitor cocktail (Roche), 1 mM NaF, and 2 mM Na_3_VO_4_ on ice for 30 min. Lysates were centrifuged at 14,000× *g* for 10 min at 4°C, and then protein content was determined by the bicinchoninic acid method using BSA as a standard. Then, 50 µg of the protein samples were separated on 4–12% sodium dodecyl sulfate (SDS)-polyacrylamide gel and then transferred to a nitrocellulose membrane. The membrane was blocked (Tris-HCl buffer, pH 7.0, 5% Tween 20 (TBST) and 5 % nonfat dried milk) for 1 h at room temperature, then probed overnight at 4 °C with primary antibodies anti-iNOS or anti β-actin (diluted 1:1000 in TBST with 5% BSA), and then incubated for 1 h at room temperature using secondary antibodies conjugated with alkaline phosphatase (1:10,000 in TBST with 5% BSA) according to the standard procedure. Immunocomplexes were visualized using an NBT/BCIP substrate and analyzed in the Molecular Imager ChemiDoc MP Imaging System (Bio-Rad, Hercules, CA, USA).

### 4.19. Statistical Analysis

Statistical analysis was performed using the software package Statistica 6 by StatSoft. Results are presented as mean ± SD. Data were analyzed using Student’s *t* test. A value of *p* ≤ 0.05 was considered statistically significant.

## 5. Conclusions

In the current study, the recombinant YGP40 protein was functionally expressed in the *Escherichia coli* system, and the obtained yield was 1.125 mg/L. Bioactivity analysis of the recombinant protein indicated that it exhibits immunomodulatory activity, in terms of cytokine and nitric oxide induction, at a level comparable to that of the natural yolkin polypeptide complex obtained from hen egg yolk. Moreover, no cytotoxic effects of rYGP40 on the BMDM cell line were observed. Our results showed that rYGP40 is not a hazard to those cells, is biologically active, and possesses significant immunoregulatory activity. Additionally, our study indicates that recombinant YGP40 from *E. coli* could be used as a robust and effective resource for further investigation of the explanation of the mechanisms of YGP40 processing and maturation and their role in the immunomodulation of the immune system.

## Data Availability

Not applicable.

## References

[1] Christmann J.L., Grayson M.J., Huang R.C. (1977). Comparative study of hen yolk phosvitin and plasma vitellogenin. Biochemistry.

[2] Anton M. (2013). Egg yolk: Structures, functionalities and processes. J. Sci. Food Agric..

[3] Zambrowicz A., Dąbrowska A., Bobak Ł., Szołtysik M. (2014). Egg yolk proteins and peptides with biological activity. Adv. Exp. Med..

[4] Mine Y., Kovaks-Nolan J. (2016). New insights in biologically active proteins and peptides derived from hen egg. Poult. Sci. J..

[5] Li H., Zhang S. (2017). Function of vitellogenin in eggs. Results Probl. Cell Differ..

[6] Yamamura J., Adachi T., Aoki N., Nakjima H., Nakamura R., Matsuda T. (1995). Precursor-product relationship between chicken vitellogenin and the yolk proteins: The 40 kDa yolk plasma glycoprotein is derived from *C* terminal cysteine-rich domain of vitellogenin II. Biochim. Biophys. Acta.

[7] Polanowski A., Zabłocka A., Sosnowska A., Janusz M., Trziszka T. (2012). Immunomodulatory activity accompanying chicken egg yolk immunoglobulin Y. Poultr. Sci..

[8] Polanowski A., Sosnowska A., Zabłocka A., Janusz M., Trziszka T. (2013). Immunologically active peptides that accompany hen egg yolk immunoglobulin Y: Separation and identification. Biol. Chem..

[9] Janusz M., Zabłocka A. (2010). Colostral proline-rich polypeptides–immunoregulatory properties and prospects of therapeutic use in Alzheimer’s disease. Curr. Alzheimer Res..

[10] Zambrowicz A., Zabłocka A., Bobak Ł., Macała J., Janusz M., Polanowski A., Trziszka T. (2017). A simple and rapid method of isolation of active polypeptide complex, yolkin, from chicken egg yolk. Food Chem..

[11] Kazana W., Mitkiewicz M., Ochnik M., Sochocka M., Zambrowicz A., Piechowiak G., Macała J., Miernikiewicz P., Zabłocka A. (2020). Yolkin isolated from hen egg yolk as a natural immunoregulator, activating innate immune response in BMDM macrophages. Oxid. Med. Cell. Longev..

[12] Zabłocka A., Zambrowicz A., Macała J., Kazana W., Polanowski A. (2018). Yolkin—a polypeptide complex isolated from chicken egg yolk with potential neuroprotective and antioxidative activity. Neuropsychiatry.

[13] Hryniewicz A., Bialuk I., Kamiński K.A., Winnicka M.M. (2007). Impairment of recognition memory in interleukin-6 knock-out mice. Eur. J. Pharm..

[14] Sterneck E., Kaplan D.R., Johnson P.F. (1996). Interleukin-6 induces expression of peripherin and cooperates with Trk receptor signaling to promote neuronal differentiation in PC12 cells. J. Neurochem..

[15] Calabrese F., Rossetti A.C., Racagni G., Gass P., Riva M.A., Molteni R. (2014). Brain-derived neurotrophic factor: A bridge between inflammation and neuroplasticity. Front. Cell. Neurosci..

[16] Waterhouse E.G., Xu B. (2009). New insight into the role of brain-derived neurotrophic factor in synaptic plasticity. Mol. Cell. Neurosci..

[17] Lemieszewska M., Jakubik-Witkowska M., Stańczykiewicz B., Zambrowicz A., Zabłocka A., Polanowski A., Trziszka T., Rymaszewska J. (2016). Pro-Cognitive properties of the immunomodulatory polypeptide complex. Yolkin, from chicken egg yolk and colostrum-derived substances: Analyses based on animal model of age-related cognitive deficits. Arch. Immun. Ther. Exp..

[18] Zabłocka A., Sosnowska A., Urbaniak A., Janusz A., Polanowski A. (2014). Peptides accompanying chicken egg yolk IgY - alternative methods of isolation and immunoregulatory activity. Food Funct..

[19] Brubaker A.L., Palmer J.L., Kovacs E.J. (2011). Age-related dysregulation of inflammation and innate immunity. Lessons learned from rodent models. Aging Dis..

[20] Shapouri-Moghaddam A., Mohammadian S., Vazini H., Taghadosi M., Esmaeili S.A., Mardani F., Seifi B., Mohammadi A., Afshari J.T., Sahebkar A. (2018). Macrophage plasticity, polarization, and function in health and disease. J. Cell Physiol..

[21] Shaw A.C., Goldstein D.R., Montgomery R.R. (2013). Age-dependent dysregulation of innate immunity. Nat. Rev. Immunol..

[22] Bradford M.M. (1976). A rapid sensitive method for the quantification of microgram quantities of protein utilizing the principle of protein-Dye Binding. Anal. Biochem..

[23] Elkin R.G., Freed M.B., Danetz S.A.H., Bidwell C. (1995). Proteolysis of Japanese quail and chicken plasma apolipoprotein B and vitellogenin by catepsin D: Similarity of resulting protein fragments with egg yolk polypeptidesd. Comp. Biochem. Physiol..

[24] Watanabe S., Alexander M., Misharin A.V., Budinger G.R.S. (2019). The role of macrophages in the resolution of inflammation. J. Clin. Investig..

[25] Dugue G.A., Descoteaux A. (2014). Macrophage cytokines Involvement in immunity and infection diseases. Front. Immunol..

[26] Inglot A.D., Janusz M., Lisowski J. (1996). Colostrinine: A proline-rich polypeptide from ovine colostrum is a modest cytokine inducer in human leukocytes. Arch. Immun. Ther. Exp..

[27] Thurm C.W., Halsey J.F. (2005). Measurement of cytokine production using whole blood. Curr. Prot. Immunol..

[28] Zambrowicz A., Zabłocka A., Sudoł M., Bobak Ł., Sosicka P., Trziszka T. (2018). The effect of carbohydrate moieties on immunoregulatory activity of yolkin polypeptide naturally occuring in egg yolk. LWT-Food Sci. Technol..

[29] Gasteiger G., D’Osulado A., Schubert D.A., Weber A., Bruscia E.M., Hartl D. (2017). Cellular innate immunity: An old game with new players. J. Innate Immun..

[30] Cummmings R.D. (2017). Stuck on sugars: How carbohydrates regulate cell adhesion, recognition, and signaling. Glycoconj. J..

[31] Moremen K.W., Tiemeyer M., Nairn A.V. (2012). Vertebrate protein glycosylation: Diversity, synthesis and function. Nat. Rev. Mol. Cell Biol..

[32] Sheng L., He Z., Chen J., Liu Y., Ma M., Cai Z. (2017). The impact of N-glycosylation on conformation and stability of immunoglobulin Y from egg yolk. Int. J. Biol. Macromol..

[33] Sambrook J.F., Russell D.W. (2001). Molecular Cloning: A Lab. Manual.

[34] Laemmli U.K. (1970). Cleavage of structural proteins during the assembly of the head of bacteriophage T4. Nature.

[35] Kumar P.D., Krishnaswamy S. (2005). Overexpression, refolding, and purification of the major immunodominant outer membrane porin OmpC from *Salmonella typhi: Characterization* of refolded OmpC. Prot. Exp..

[36] Mosmann T. (1983). Rapid colorimetric assay for cellular growth and survival: Application to proliferation and cytotoxicity assays. J. Immunol. Methods.

[37] Guevara I., Iwanejko J., Dembińska-Kieć A., Pankiewicz J., Wanat A., Anna P., Gołąbek I., Bartuś S., Malczewska-Malec M., Szczudlik A. (1998). Determination of nitrite/nitrate in human biological material by the simple Griess reaction. Clin. Chim. Act..

